# Phenotypic Characterization and Transformation Attempts Reveal Peculiar Traits of *Xylella fastidiosa* Subspecies *pauca* Strain De Donno

**DOI:** 10.3390/microorganisms8111832

**Published:** 2020-11-20

**Authors:** Giusy D’Attoma, Massimiliano Morelli, Leonardo De La Fuente, Paul A. Cobine, Maria Saponari, Alessandra Alves de Souza, Angelo De Stradis, Pasquale Saldarelli

**Affiliations:** 1 Consiglio Nazionale delle Ricerche, Istituto per la Protezione Sostenibile delle Piante, Sede Secondaria di Bari, 70124 Bari, Italy; giusy.dattoma@ipsp.cnr.it (G.D.); massimiliano.morelli@ipsp.cnr.it (M.M.); maria.saponari@ipsp.cnr.it (M.S.); angelo.destradis@ipsp.cnr.it (A.D.S.); 2 Department of Entomology and Plant Pathology, Auburn University, Auburn, AL 36849, USA; lzd0005@auburn.edu; 3 Department of Biological Sciences, Auburn University, Auburn, AL 36849, USA; pac0006@auburn.edu; 4 Centro de Citricultura Sylvio Moreir Agronomic Institute (IAC), Cordeirópolis 13490-970, SP, Brazil; desouza@ccsm.br

**Keywords:** *Xylella fastidiosa* strain De Donno, biofilm formation, type I restriction–modification systems, natural competence, green fluorescent protein, twitching motility, *rpfF* gene, cell-to-cell aggregation

## Abstract

*Xylella fastidiosa* subsp. *pauca* strain De Donno has been recently identified as the causal agent of a severe disease affecting olive trees in a wide area of the Apulia Region (Italy). While insights on the genetics and epidemiology of this virulent strain have been gained, its phenotypic and biological traits remained to be explored. We investigated in vitro behavior of the strain and compare its relevant biological features (growth rate, biofilm formation, cell–cell aggregation, and twitching motility) with those of the type strain Temecula1. The experiments clearly showed that the strain De Donno did not show fringe on the agar plates, produced larger amounts of biofilm and had a more aggregative behavior than the strain Temecula1. Repeated attempts to transform, by natural competence, the strain De Donno failed to produce a GFP-expressing and a knockout mutant for the *rpfF* gene. Computational prediction allowed us to identify potentially deleterious sequence variations most likely affecting the natural competence and the lack of fringe formation. GFP and *rpfF*- mutants were successfully obtained by co-electroporation in the presence of an inhibitor of the type I restriction–modification system. The availability of De Donno mutant strains will open for new explorations of its interactions with hosts and insect vectors.

## 1. Introduction

*Xylella fastidiosa* (*X. fastidiosa*) is a Gram-negative bacterium in the family Xanthomonadaceae that is able to colonize the xylem network of over 500 species of host plants [1] and the foregut of hemipteran xylem-feeding insects, belonging to Cicadellidae Cicadellinae (sharpshooters) and Cercopoidea (spittlebugs) [2]. Many economically relevant diseases are caused by the bacterium, including Pierce’s disease (PD) of grapevine, citrus variegated chlorosis (CVC), leaf scorch of coffee, plum and almond [3], and olive quick decline syndrome (OQDS), the latter discovered in 2013 in olive groves in the south of the Apulia region, in Italy [4,5] and now representing a devastating phytosanitary emergency severely affecting, according to a recent estimate, 4 million olive trees [6].

To date, the molecular mechanisms behind *X. fastidiosa* pathogenicity are still unclear. Current knowledge shows that *X. fastidiosa*-associated disease symptoms, as leaf scorching and stunted growth, can be mostly explained by the progressive and increasing inability of infected hosts to mobilize water and nutrients from the roots to the leaves, due to the extensive colonization of the xylem vessels and their blockage by bacterial aggregates and exopolymeric substances (EPS) deposition, in conjunction with plant tyloses and gum [7,8]. Several findings corroborate the hypothesis that the virulence of the pathogen relies on a fine balance between more motile bacterial forms, able to efficiently multiply and move within and between the xylem conduits, and sticky aggregative cells forming a biofilm, which are responsible for the vessels clogging and insects acquisition [9,10]. This dual lifestyle behavior arises from a cell density-dependent regulation, otherwise known as quorum sensing, based on the production and perception of an extracellular signal molecule named diffusible signal factor (DSF) [9,11]. DSF is an unsaturated fatty acid [12], produced by the *rpfF* gene, that initiates a transduction cascade resulting in up- or downregulation of several virulence genes required for adhesiveness, biofilm formation, and motility [13].

*X. fastidiosa* lacks flagella, and the process by which the pathogen spreads from the infection site and moves from one location to another to colonize the entire plant is not entirely elucidated. The movement of *X. fastidiosa* cells is an active process and seems to depend on their ability to enzymatically breach pit membranes, which, by interconnecting vessels, allow flow sap passage and hinder pathogen movement [14,15]. However, an *X. fastidiosa* upstream migration, via twitching motility, using long type IV pili was demonstrated [16], thus explaining downward translocation in the plant. The twitching motility is a type of flagella-independent bacterial translocation over moist surfaces [17], which occurs through extension, binding, and retraction of type IV pili. In vitro twitching motility proved to be associated with the development of a “peripheral fringe” around *X. fastidiosa* colonies grown on a solid medium, which is due to ‘rafts’ of aggregated cells that move away from the colony [18,19].

Several genes encoding for various surface-associated, fimbrial and afimbrial adhesin proteins were found to form the basis of biological traits important for *X. fastidiosa* virulence such as adhesiveness, intraplant migration, host colonization, and biofilm formation [20,21,22]. Nevertheless, the availability of water and mineral nutrients, which are naturally dispersed in xylem sap, can act as a stimulus, eliciting changes in bacterial cells that can modulate the establishment and development of biofilm [23,24].

The host range and pathogenicity of different *X. fastidiosa* strains and subspecies vary greatly [25]. *X. fastidiosa* is naturally competent in the uptake of DNA fragments from the external environment and is able to incorporate them into the genome by homologous recombination [26]. This biological trait has an evolutionary significance since it could provide an efficient route for horizontal gene transfer and confer a fitness benefit [27,28], allowing *X. fastidiosa* to explore and adapt to a new environment [29]. Some studies have shown that homologous recombination plays a significant role in generating genetic diversity, potentially more than point mutations [30,31]. Recombination has been shown to increase the rate of pathogen adaptation [32] and to lead to the emergence of new diseases [33,34,35,36].

Natural competence of *X. fastidiosa* has already been shown in vitro for strains belonging to subspecies *fastidiosa* and *multiplex* [26,37,38]. However, recent investigations have shown that natural competence rates are different among strains [37] and several factors may affect *X. fastidiosa* transformation efficiency [27]. Cell density does not directly influence the transformation rate, while the growth stage was shown to be one of the many factors regulating *X. fastidiosa* competence [26] and recombination efficiency can be also strongly modulated by the sequence similarity and the size of transforming DNA [39]. Analysis of the genome of several strains revealed also that several potential restriction–modification (R–M) systems are present in *X. fastidiosa* [40,41,42]. These systems may play a role in processing incoming DNA at specific sequence locations, once modified by a cognate methyltransferase [43]. Particularly, restriction by the type I systems has been shown to have a major impact on the stable acquisition of foreign DNA [44]. Likewise, it has been found that twitching motility is significantly correlated with natural transformation and recombination efficiency [27,37].

Although several studies have been done on *X. fastidiosa* strains prevalent in the Americas, so far limited data are available on phenotypic characterization of emerging European strains [45,46,47]. This study aims to contribute to filling this gap focusing on phenotypic and biological traits of the *X. fastidiosa* subsp. *pauca* strain De Donno, the etiological agent of the OQDS. Relevant biological features (growth rate, biofilm formation, cell–cell aggregation, and twitching motility) of this strain were in vitro assessed and compared to those of *X. fastidiosa* Temecula1, the reference strain of the subspecies *fastidiosa*, isolated from grapevine in California. Transformation protocols have been explored to test the natural competence ability of the olive strain and its suitability for genetic manipulation, which will be useful for developing bacterial mutants as a functional tool to gain more insights about the interactions of this strain with either the vector and its plant hosts.

## 2. Materials and Methods

### 2.1. Bacterial Strains and Culture Conditions

*X. fastidiosa* subsp. *fastidiosa* strain Temecula1 [48] and subsp. *pauca* strain De Donno [49] were used in this study. Both strains were cultured at 28° for five days in periwinkle wilt (PW) [50] agar plates without the addition of phenol red [38], before further use. When needed culture medium was supplemented with 50 µg/mL kanamycin for antibiotic selection.

### 2.2. Growth Curve, Biofilm, Settling Rate, and Twitching Motility Measurements of X. fastidiosa Strains

Growth curves were generated by culturing strains in PD3 [51] liquid medium in 96-well plates and measuring the optical density at a wavelength of 600 nm (OD_600_) every day for 7 days. At the beginning of the experiment (day 0), each well was inoculated with 190 µL of medium and 10 µL of five-day-old cell suspension (OD_600_ = 0.5). Eight wells per strain were used in repetition, while additional eight wells were filled with 200 µL of medium as control. The 96-well plates were incubated at 28 °C, with orbital shaking at 150 rpm. OD_600_ was measured by Cytation 3 Multi-Mode imaging reader (BioTek, Winooski, VT, USA) and the resulting values were subtracted from those of control wells. The log-transformed growth rates were estimated based on the slope of the growth curve calculated at the exponential phase (about 1–4 days), according to the formula by Tyson et al. [8]:

$$\mathrm{rate}=\frac{\left[\ln\left({\mathrm{OD}}_{600}\;\mathrm{day}\;4\right)-\ln\left({\mathrm{OD}}_{600}\;\mathrm{day}\;1\right)\right]}{\mathrm{time}}$$

Biofilm was quantified using the crystal violet assay [24], at the end of the growth curve experiment (day 7). Briefly, the supernatant was transferred to a new 96-well plate, which was read at 600 nm to evaluate the planktonic cell fraction. The original plate was washed three times with water before adding a 0.1% solution of crystal violet to each well. After 15 min the crystal violet solution was discarded and the plate was washed three times with water. Each well was filled with absolute ethanol and the plate was incubated for 20 min to dissolve the crystal violet. The plate was then read at 600 nm to evaluate the fraction of cells adhering to the wells to form a biofilm. Each experiment was repeated three times.

Settling rate, an index of cell-to-cell aggregation, was determined by measuring the reduction of absorbance at 600 nm of a cell suspension, due to the sedimentation of cell aggregates. Five-day-old cells (OD_600_ = 1.0) were collected from a PD3 agar plate and suspended in 1 mL PD3 medium. The suspension was vigorously homogenized by pipetting and cuvettes placed into a spectrophotometer, where the OD_600_ values were continuously measured for 3 min. The settling rate was automatically generated by the instrument and calculated as the slope of the linear portion of the decreasing change in the OD_600_ over time [37].

Twitching motility was assessed on PW and PD3 agar plates. Five-day-old colonies were scraped from the agar plates and were spotted in quadruplicate onto fresh agar plates. The colony peripheral fringe morphology was examined after 48 h of incubation at 28 °C, under an Eclipse Ti inverted microscope (Nikon, Melville, NY, USA), using phase contrast. All the experiments were repeated independently at least three times with at least three technical replicates per time.

#### Statistical Analysis

Statistical analyses of quantitative data relative to biofilm formation, growth, and settling rates were performed in R environment, using RStudio version 1.2.5033 [52]. Data normal distribution and homogeneity of variances were assessed according to the Shapiro–Wilk’s and to Levene’s tests, respectively [53]. Statistical significance of mean differences was evaluated by a Student’s *t*-test at the 0.05 alpha level. The R ggplot2 package [54] was used to generate the graphic plots.

### 2.3. Transformation Protocols

Transformations of *X. fastidiosa* subspecies *pauca* strain De Donno were carried out to develop a mutant expressing a green fluorescence protein and a knock-out strain for the *rpfF* gene.

#### 2.3.1. Plasmid Constructs

The pKLN59, a replicating plasmid developed by [55] was used for the stable expression of a green fluorescent protein in the strain De Donno. In particular, the pKLN59 is an allelic exchange plasmid designed for DNA integration, into the bacterial chromosome, of an artificial operon in which the GFP gene is fused with the gene for kanamycin resistance. The allelic exchange is designed to occur within an untranscribed region of the chromosomal 23S ribosomal RNA gene. We preliminary ascertained by BLASTn analysis that the nucleotide sequences of the fragments of the *X. fastidiosa* Temecula1 genome, carried by the pKLN59 plasmid, share a 99% identity with the corresponding regions of the De Donno genome.

**Figure 1:**
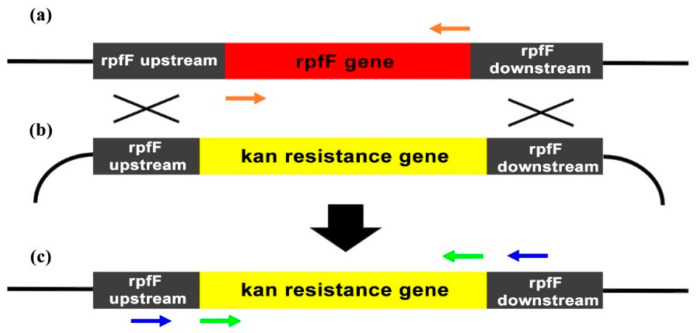
Schematic representation of the mutagenesis cassette cloned in the donor plasmid prpfF-kan for homologous recombination and replacement of the target *rpfF* gene with the kanamycin resistance gene (*kan*) in the genome of *X. fastidiosa* strain De Donno. (**a**) *rpfF* gene structure on the *X. fastidiosa* gene region, indicating the two (*rpfF* upstream and *rpfF* downstream) amplified regions of the gene; (**b**) structure of the prpfF-kan donor plasmid structure; and (**c**) structure of the *X. fastidiosa* genome after homologous recombination. PCR primers RpfF_F/RpfF_R, Kan_F/Kan_R, RpfFMut_F/RpfFMut_R, used to verify the effectiveness of recombination, are indicated by orange, green, and blue arrows, respectively.

The protocol described by Kandel et al. [56] was used to develop the knockout template for the *rpfF* gene mutant. To construct the mutagenesis cassette, two approximately 800 bp long upstream and downstream regions of the *rpfF* gene were amplified from DNA extracted from the strain De Donno using primer pairs Up_F/Up_R and Down_F and Down_R. Kanamycin resistance gene was amplified from the DNA plasmid pUC4K (GE Healthcare, Chicago, IL, USA) using primers Kan_F and Kan_R., Primers Up_R and Down_F were 5′ tailed with a fragment of 21 bp homologous to the antibiotic cassette, thus allowing fusion of the upstream and downstream genomic regions to the *kan* resistance gene by overlap PCR. The three separate amplicons (upstream *rpfF*, *kan* gene, and downstream *rpfF*) were excised from agarose gel and used in an overlap extension PCR using the terminal primers Up_F and Down_R. The resulting product, harboring the *kan* resistance gene flanked by the upstream and downstream regions of the target *rpfF* gene, was gel-purified and T/A cloned into pSC-A-amp/kan cloning vector (Agilent Technologies, Santa Clara, CA, USA) generating the plasmid prpfF-kan (Figure 1). The correctness of the mutagenesis cassette was verified by *ClaI* restriction (NEB, Ipswich, MA, USA) and further confirmed by sequencing with the M13 universal primer pair.

#### 2.3.2. Electroporation

Plasmids pKLN59 and prpfF-kan were introduced into the *X. fastidiosa* strain De Donno by electroporation, based on the procedure described by Newman et al. [55]. A 10^6^/^mL^ suspension of the recipient strain was prepared in 10% sterile glycerol and grown on buffered charcoal yeast extract (BCYE) agar plates [57]. Electrocompetent cells were freshly prepared for each transformation by resuspending colonies from 10-day-old plates in 10% chilled glycerol. Cells were washed by pelleting and resuspending three times with 10% glycerol before final resuspension in 40 µL of 10% sterile glycerol. Solutions and centrifugations were carried out at 4 °C. About 5 µg of each plasmid in a volume of 10 µL were electroporated into 40 µL of electrocompetent *X. fastidiosa* cells, with and without the TypeOne™ Restriction Inhibitor (5 µg; Lucigen, Middleton, WI, USA), in a 0.1 cm-gap cuvette at 1.8 kV, 200 Ω, and a capacitance of 25 µF in a GenePulser (Bio-Rad, Hercules, CA, USA) apparatus. Electroporated cells were recovered for 24 h in PW broth, plated on selective BCYE agar medium supplemented with kanamycin and incubated at 28 °C for 7–21 days.

The colonies resulting from the transformation with the pKLN59 plasmid were restreaked onto fresh selective plates five times before checking the cell fluorescence. At 10-day intervals, the colonies were restreaked again onto a non-selective medium and further scored for fluorescence, to assess the stability of GFP expression. The chromosomal integration of the *kan*-*gfp* cassette was assessed by PCR, using a primer set straddling the recombination site. The forward primer Int_F was designed within the pKLN59 *kan* resistance gene, and the reverse primer Int_R within the genomic portion downstream of the exchange site, located, as mentioned above, in an untranscribed region of the 23S ribosomal RNA gene.

Similarly, colonies resulting from the transformation with prpfF-kan plasmid were selected by multiple streaking onto PD3 agar plates containing kanamycin. The genomic replacement of the target *rpfF* gene by the antibiotic-resistant gene was confirmed by PCR by using Kan_F/Kan_R, RpfF_F/RpfF_R, and RpfFMut_F/RpfFMut_R primer pairs. *X. fastidiosa* transformants obtained with pKLN59 and prpfF-kan plasmids were named DD*gfp*, and DD*rpfF-*, respectively.

#### 2.3.3. Observation of Fluorescence

GFP-expressing cells were observed using an Eclipse 80i Fluorescence Microscope equipped with a VideoConfocal (ViCo) system (Nikon Corporation, Tokyo, Japan). For this purpose, five-day-old cell cultures of *X. fastidiosa* mutant strain DD*gfp* were scraped from BCYE agar plates and resuspended in sterile phosphate-buffered saline (PBS) solution. Fluorescent images were captured through a bandpass ET/GFP cube filter (Nikon Corporation, Japan) (excitation at 440–470 nm, emission at 525–550 nm) and analyzed with Image-Pro Plus 6.0 software (Media 11 Cybernetics, Rockville, MD, USA). Adobe Photoshop CS6 software (Adobe Systems, Inc., San Jose, CA, USA) was used to assemble and label the final figures.

#### 2.3.4. Natural Competence

The natural transformation was attempted, based on the protocol described by Kandel et al. [56], in PD3 agar plates by using plasmids pKLN59 and prpfF-kan. The strain De Donno was adjusted to OD_600_ of 0.25 and 0.5 in PD3 broth. Ten microliters of this suspension were spotted onto PD3 agar plates, and the spots were allowed to dry for 1 h. About 2 µg of each plasmid in 10-µL volume were added, separately, to the spots. Following incubation at 28 °C for about three days, spots were suspended in 1 mL of PD3 and serial dilutions were cultured in PW agar plates supplemented with kanamycin, in triplicates. After two to three weeks of incubation at 28 °C, plates were checked for viable cells. Spots without the addition of plasmids were included as controls.

#### 2.3.5. DNA Extraction

For DNA extraction, five-day-old cell cultures of *X. fastidiosa* De Donno wild type or mutant strains were scraped from PW or PD3 agar plates and resuspended in 500 µL of PBS medium. DNA was extracted using hexadecyltrimethylammonium bromide (CTAB) buffer, according to the standard procedure described by Loconsole et al. [58]. Purified plasmid DNA was obtained using the Plasmid DNA Mini Prep kit (Fisher Molecular Biology, Trevose, PA, USA).

#### 2.3.6. PCR Amplification and Primer Design

All the primer sets mentioned in this study, when not otherwise specified, were designed with the NCBI Primer-Blast tool [59] and are reported in Table 1. PCR reactions were performed with a standard protocol using Phusion^®^ High-Fidelity DNA Polymerase (Thermo Fisher Scientific, Waltham, MA, USA) for long template cloning or DreamTaq DNA Polymerase (Thermo Fisher Scientific, USA) for standard amplifications. PCR products were gel purified using the MicroElute gel/PCR purification kit (Fisher Molecular Biology, USA).

#### 2.3.7. Analysis of Restriction–Modification Systems

Bioinformatic screening of the genome sequence of *X. fastidiosa* De Donno to investigate the presence of putative R–M systems was performed using tools available in the Restriction Enzyme Database (REBASE) [40,60], which allows extensive analysis of the R–M systems that are predicted to be present in bacterial and archaeal genomes available from GenBank.

#### 2.3.8. Analysis of Sequence Variations in Genes Involved in Natural Competence and Twitching Motility

A comparison between the sequences of genes known to be responsible for natural competence and twitching motility was made between the genomes of the strain De Donno (CP020870.1) and the strain Temecula1 (NC_004556). The sequences of the genes *pilA-1*, *pilB*, *pilM*, *pilQ*, *pilO*, *recA*, *comA*, and *comF* were retrieved from the GenBank database using the Ensembl Bacteria browser [61] and their protein products compared in a pairwise alignment, using the BLASTp tool [62].

Resulting mutations were submitted to the PROVEAN (Protein Variation Effect Analyzer, JCVI, La Jolla, CA, USA) web server [63], in order to predict whether detected amino acid substitutions or indels could have an impact on the biological function of the proteins. Based on a homology-based approach, this computational tool accepts a protein sequence and amino acid variations as input, performs a BLAST search to identify homologous sequences, and generates a score, with a default cut-off value of −2.5 [64]. Mutations found in De Donno gene products that returned a PROVEAN score above this threshold were predicted as neutral, whereas scores below or equal to −2.5 were classified as deleterious.

## 3. Results

### 3.1. Phenotypic Differences between X. fastidiosa Strains De Donno and Temecula1

#### 3.1.1. Growth Rate

**Figure 2:**
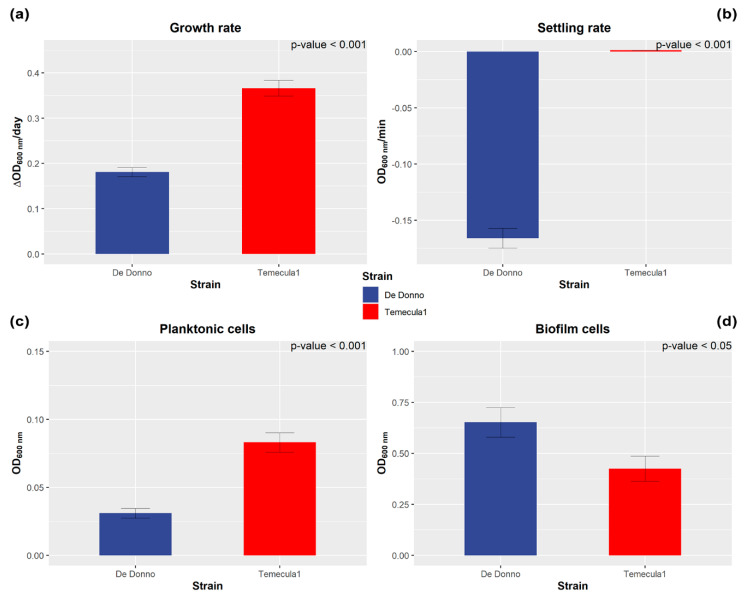
Comparison of biological traits between *X. fastidiosa* strains Temecula1 and De Donno. Both strains were grown in the PD3 medium. The growth rate (**a**) was calculated from the growth curve at the exponential phase (1–4 dpi). Settling rate (**b**) was assessed by suspending bacterial culture in a 1 mL-cuvette and continuously measuring optical density for 3 min. Planktonic growth (**c**) was assessed by measuring optical density (OD) at the end of the growth-curve experiment (7 dpi). Biofilm formation (**d**) was assessed by measuring OD, using crystal violet staining in the 96-well plates, at the end of the growth-curve experiment (7 dpi). Experiments (**a**–**d**) were repeated independently three times. For 96-well plate assays (**a**,**c**,**d**) 6 wells per plate and 3 plates per experiment were used for each strain. Error bars indicate the standard error of the mean. OD was measured at 600 nm wavelength. *p*-values show statistically significant differences between strains, as assessed by a Student’s *t*-test at the 5% significance level.

The growth rate measured at the exponential growth phase proved to be different between *X. fastidiosa* strains Temecula1 and De Donno. As shown in Figure 2a, Temecula1 showed a mean growth rate (0.37 ΔOD_600_/day) significantly (*p*-value < 0.001) higher than De Donno, whose cell populations reached lower optical density values (0.18 ΔOD_600_/day). However, the observed growth values could be affected by interference from cell aggregates that may have adhered to the surface of the plate walls or be deposited at the bottom of the wells.

#### 3.1.2. Settling Rate

**Figure 3:**
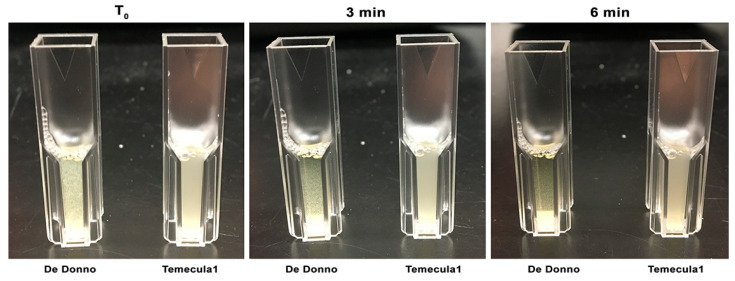
Comparison of precipitation behavior between *X. fastidiosa* strains Temecula1 and De Donno used in this study. Bacterial cells were suspended in a 1 mL-cuvette in the PD3 medium and allowed settling for 6 min. Strain De Donno showed reduced turbidity, index of a faster settling, already visible after 3 min.

Analysis of cell settling rates, as a measurement of the decrease observed in the optical density due to aggregates that settled on the bottom of a cuvette, demonstrated that De Donno cells had a more aggregative phenotype, showing significantly higher (*p*-value ≤ 0.001) sedimentation rates (mean = 0.16 OD_600_/min) than Temecula1 cells (mean = 0.001 OD_600_/min; Figure 2b). This observation showed the ability of the strain De Donno to make self-aggregation, sometimes described as self-agglutination, meaning the propensity of its cells to bind to each other, thus forming clumps that may adhere to the surfaces, resulting in the first step of biofilm formation, or can also remain planktonic [65,66]. This different behavior with respect to strain Temecula1 was further confirmed by macroscopical observations. As shown in Figure 3, the turbidity of cell suspensions changed considerably in De Donno after 3 min of incubation, whereas Temecula1 cell suspensions were not only more turbid at T_0_, but maintained the same turbidity over time, a feature that is usually associated with planktonic growth [67]. In fact, the optical density of Temecula1 cell culture (mean = 0.083 OD_600_), measuring the growth of supernatant fractions (i.e., ‘planktonic cells’) after seven days of incubation, was significantly (*p*-value ≤ 0.001) higher than strain De Donno (mean = 0.031 OD_600_), resulting almost three times higher (Figure 2c).

#### 3.1.3. Biofilm Formation

The in vitro growth under orbital agitation of the two strain led to the production of a “ring” of bacterial cells attached on the sidewalls of the well, at the air–liquid interface, that is known to constitute *X. fastidiosa* biofilm as it presents cell aggregates surrounded by an abundant exopolymeric matrix [67]. When this cell ring was stained with crystal violet, we observed a significantly (*p*-value ≤ 0.05) higher tendency of the strain De Donno (mean = 0.652 OD_600_) to form biofilm than Temecula1 cells (mean = 0.0425 OD_600_; Figure 2d).

#### 3.1.4. Twitching Motility

**Figure 4:**
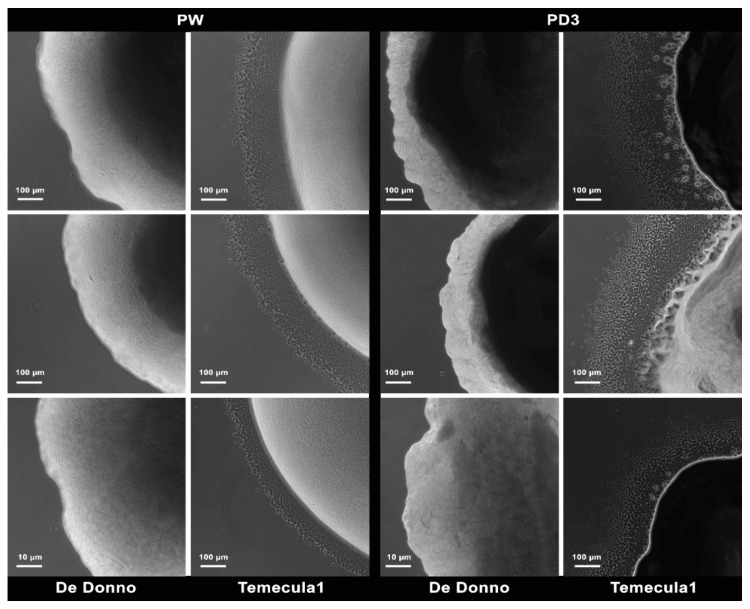
Twitching motility of *X. fastidiosa* strains Temecula1 and De Donno used in this study, assessed by measuring the fringe width of the colonies spotted on PW (**left**) or PD3 (**right**) plates 48 h post-inoculation. Measurements included three biological replicates with at least five technical replicates each.

We observed that the colonies of the strain De Donno lacked the fringe associated with the bacterial ability to twitch on solid surfaces. The same phenotype occurred both on modified PW and PD3 plates after 48 h of growth. On the contrary, under the same experimental conditions, the colonies of strain Temecula1 spotted on both media produced remarkable fringes, with dimensions comparable to those observed in other studies [38,56] (Figure 4).

### 3.2. Attempts to Transform X. fastidiosa Strain De Donno

#### 3.2.1. Transformation by Electroporation

We attempted to transform the *X. fastidiosa* subsp. *pauca* strain De Donno to get: (i) a recombinant strain harboring a *gfp* gene, to allow tracking by fluorescence microscopy, and (ii) a mutant strain lacking *rpfF*, a key gene involved in *quorum-sensing* regulation. A first attempt of electrotransformation of *X. fastidiosa* De Donno with 5 µg of plasmid DNA pKLN59 and prpfF-kan, following the standard procedure described by Newman et al. [55] yielded no transformants, as no colonies were recovered on selective BCYE plates supplemented with kanamycin, indicating that neither *gfp* nor *kan*-resistance genes integrated into the recipient De Donno chromosome. At the same time, however, the complete absence of colonies, served as a control test to prove that no spontaneous resistance could arise using 50 µg/mL kanamycin in our experiments.

Accessing the Restriction Enzyme Database (REBASE), we found that similarly to other *X. fastidiosa* strains [68,69] De Donno is predicted to encode functional type I R–M systems. We identified three distinct type I R–M systems, named, according to the nomenclature proposed by O’Leary et al. [69]: Xfa1 (locus tag B9J09_11625-B9J09_11635), Xfa2 (locus tag B9J09_11645-B9J09_11660), and Xfa3 (locus tag B9J09_11710-B9J09_11720). The presence of R–M systems could have interfered with the acquisition of foreign plasmid DNA in our preliminary attempts, we proceeded with the addition of the TypeOne™ Restriction Inhibitor (Lucigen, Middleton, WI, USA) to the electroporation mixture. Following this protocol adjustment, kanamycin-resistant transformants were successfully obtained from both pKLN59 and prpfF-kan electroporations producing the mutant strains DD*gfp* and DD*rpfF-*, respectively.

#### 3.2.2. Assessment of Stable Integration of the *kan*-*gfp* Cassette into the *X. fastidiosa* De Donno Chromosome

The plasmid vector pKLN59, used to construct the green-fluorescent strain DD*gfp*, was originally designed to integrate the *kan*-*gfp* cassette into the bacterial chromosome by double homologous recombination in a region located within the ribosomal 23S gene and immediately downstream from a strong promoter [55]. Therefore, if integrated, the gene cassette is controlled by a native *X. fastidiosa* promoter, constitutively and stably expressed over time.

**Figure 5:**
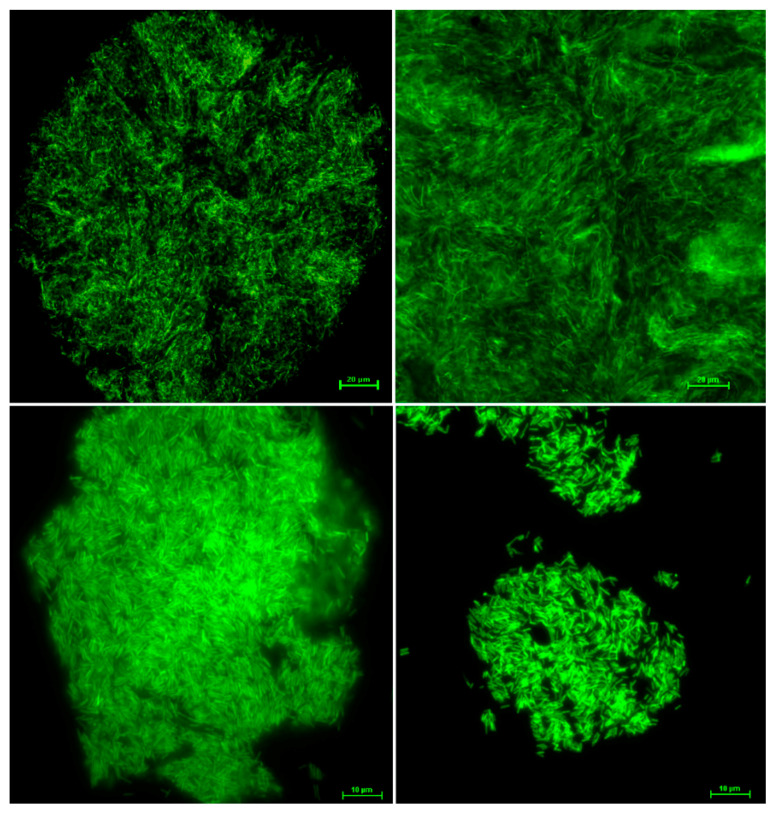
Fluorescence microscopy observations of *X. fastidiosa* strain De Donno transformed with the pKLN59 plasmid harboring the green fluorescent protein (GFP) gene. Cell aggregates were collected from buffered charcoal yeast extract (BCYE) agar plates supplemented with kanamycin, after five rounds of restreaking.

To assess the stability of the *kan*-*gfp* cassette, cell cultures of the strain DD*gfp* were restreaked five times onto selective medium. Bright green fluorescent cells aggregated in their biofilm extracellular matrix were observed under a fluorescence microscope (Figure 5). DD*gfp* strain showed the same fluorescent phenotype after repeated rounds of restreaking onto a non-selective medium, and no reversion was observed, thus confirming the stability of the insert and its high level of expression.

**Figure 6:**
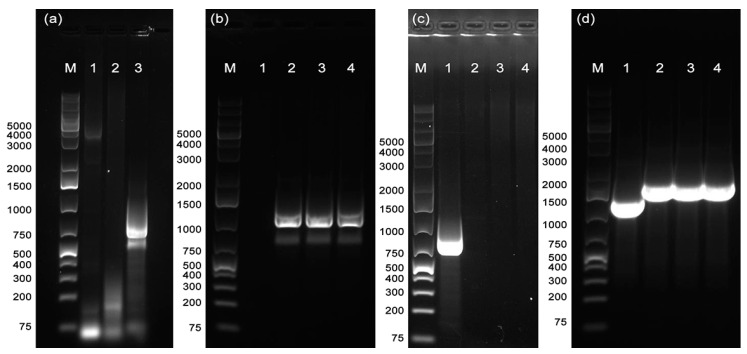
Agarose gel electrophoresis (1.2%) of PCR performed to confirm the recombination events. (**a**) Primer pair Int_F/Int_R. Lane 1: *X. fastidiosa* wild-type strain De Donno. Lane 2: pKLN59 plasmid. Lane 3: *X. fastidiosa* mutant strain DD*gfp*. (**b**) Primer pair Kan_F/Kan_R. Lane 1: *X. fastidiosa* wild-type strain De Donno. Lanes 2–4: *X. fastidiosa* mutant strain DD*rpfF*-. (**c**) Primer pair RpfF_F/RpfF_R. Lane 1: *X. fastidiosa* wild-type strain De Donno. Lanes 2–4: *X. fastidiosa* mutant strain DD*rpfF*-. (**d**) Primer pair RpfFMut_F/RpfFMut_R. Lane 1: *X. fastidiosa* wild-type strain De Donno. Lanes 2–4: *X. fastidiosa* mutant strain DD*rpfF*-. M: GeneRuler 1 kb Plus DNA Ladder (Thermo Fisher Scientific, USA).

To further assess the integration of the *kan-gfp* cassette into the De Donno genome, a PCR test was performed on DNA extracted from DD*gfp* colonies with primers Int_F and Int_R (Table 1), targeting the region encompassing the pKLN59 *kan* resistance gene and the *X. fastidiosa* genome region downstream the exchange site. PCR reactions yielded an amplicon of the predicted size (750 bp), from DD*gfp* DNA (Figure 6a, lane 3), while no amplification occurred either for the wild-type strain De Donno or for the purified pKLN59 plasmid (Figure 6a, lanes 1–2). These PCR results confirmed that the integration of the mutagenesis cassette into the bacterial chromosome occurred successfully at the expected locus.

#### 3.2.3. Assessment of Recombination in the *rpfF* Knockout Strain DD*rpfF*-

The prpfF-kan plasmid was constructed (Figure 1) to allow the substitution, by homologous recombination, of the target *rpfF* gene in the parental strain genome, with the *kan* resistance gene of the plasmid. Transformation of the strain De Donno with the prpfF-kan plasmid yielded kanamycin-resistant colonies, selected upon repeated streaking onto a selective substrate containing the antibiotic. PCR analysis was then performed on the selected colonies to confirm the integration of the cassette into the target region. An amplicon of 1203 bp, corresponding to the *kan* resistance gene, was amplified with primers Kan_F/Kan_R (Table 1) from three selected clones of the DD*rpfF*- mutant (Figure 6b, lanes 2–4), whereas primers RpfF_F/RpfF_R designed to amplify the *rpfF* gene yielded the expected 830 bp fragment from the wild-type strain only (Figure 6c, lane 1). Furthermore, the amplification with primer pair RpfFMut_F/RpfFMut_R flanking the recombination site resulted in two distinct amplicons of 1370 bp and 1697 bp, respectively, in the wild-type strain (Figure 6d, lane 1) and in the DD*rpfF*- clones (Figure 6d, lanes 2–4) harboring the inserted *kan* gene. Taken together, these results confirmed that the marker gene successfully replaced the *rpfF* target gene into the bacterial chromosome.

#### 3.2.4. Natural Transformation

Several attempts to transform the strain De Donno exploiting its natural competence failed. We were not able to recover any colony on kanamycin-supplemented plates, up to 21 days of incubation, neither using the pKLN59 nor the prpfF-kan plasmids as donors of the antibiotic resistance gene. Thus under our experimental conditions, we failed to obtain transformants, indicating that *X. fastidiosa* strain De Donno most likely is unable to uptake exogenous DNA and undergo natural competence. Conversely, the natural transformation of the strain Temecula1 with the prpfF-kan plasmid successfully yielded kanamycin-resistant colonies (data not shown).

#### 3.2.5. In Silico Prediction of the Effects of Sequence Variations in Genes Involved in Natural Competence and Twitching Motility

Sequence pairwise comparison (Temecula1 vs. De Donno) of the proteins encoded by eight genes (*pilA-1*, *pilB*, *pilM*, *pilQ*, *pilO*, *recA*, *comA*, and *comF*) predicted to be involved in the mechanisms of natural competence and twitching motility in *X. fastidiosa* [27], identified in the strain De Donno a total of 99 mutations, 92% of which were amino acid substitutions. In silico analysis performed with PROVEAN tool predicted only five substitutions to be potentially deleterious, i.e., resulting in alterations in the biological function of proteins, and the rest to be neutral (Table S1). Deleterious mutations were predicted in the genes *comA* (L400F), a transcriptional regulator involved in genetic competence (Kung and Almeida, 2014), and *pilA-1* (T95K, G104D) and *pilM* (A101V, P267S) both constituents of the extracellular part of type IV pili [70], which contributes to twitching motility and natural competence [38,71].

## 4. Discussion

Following the discovery in 2013 of OQDS in Apulia, an unprecedented research effort has been devoted to gain fundamental knowledge on its causal agent [72]. The urgency to develop strategies to control and limit the spread of the infections in the EU territory, and to provide scientific data for the implementation of containment measures and risk assessment plans, has initially oriented scientific investigations towards the study of the epidemiology, etiology, and biology of the OQDS. The description of the genome sequences of De Donno [49], and of several other European strains [47,73,74,75] paves the way to elucidate their virulence and phenotypic traits providing the necessary information to allow the generation of knockout mutants of specific genes or gene tagging [44].

Our studies have shown that *X. fastidiosa* De Donno is not naturally competent under our laboratory conditions, this is similar to what was found with the citrus-pathogenic strain 9a5c, also belonging to the subspecies *pauca* [42]. However, *X. fastidiosa* 11399, another CVC-associated strain [76], has been recently reported to be transformable [66]. The enhanced transformation efficiency of this strain could be explained by the lack of four genes of the Type I restriction–modification system, emerging from a genome comparative analysis between the two citrus-infected strains [42].

The restriction–modification systems, widely distributed among bacteria, discriminating between self and non-self DNA, may serve as a tool of defense for bacterial cells, by restricting the successful transfer and propagation of foreign DNA. The general principle of R–M systems is that the host genomic DNA is methylated at specific sequences and protected from restriction by endonucleases, whereas exogenic DNA is digested due to the lack of this modification. Ultimately these systems provide an obstacle to genetic manipulation requiring transformation with DNA from an external source [68]. Four types of R–M systems have been identified, with differences related to the enzyme composition and cofactors, the recognition sequence symmetry and the cleavage characteristics. Type I restriction enzymes are multisubunit enzymes with separate restriction (R), methylation (M), and DNA sequence-recognition (S) activities [77].

Previous transformation trials showed that type I R–M systems are particularly involved in the stable acquisition of exogenous DNA by *X. fastidiosa*, as the coelectroporation of plasmids isolated from *E. coli* into the strain Temecula1 with Type I R–M inhibitors, conferred a five-fold increase of the frequency of transformation [44]. Similarly, the type I R–M system was involved in the stable transformation of *Staphylococcus aureus*, which is notoriously difficult to genetically manipulate. Genome comparative analyses indicated that a mutation in the *Sau1* type I R–M system is associated with the enhanced ability of *S. aureus* RN4220, the only transformable strain, to accept foreign DNA [78].

REBASE analysis [40] of the *X. fastidiosa* De Donno genome has uncovered three potential R–M systems (Xfa1, Xfa2, and Xfa3). Interestingly, as highlighted in a recent study by [69], the two adjacent Xfa1 and Xfa2 are common in several *X. fastidiosa* strains, including the natural competent Temecula1, whereas, Xfa3 is conserved only in strains of the subsp. *multiplex* and *pauca*, including the non-transformable 9a5c. This genomic evidence suggested attempting the transformation by electroporation in the presence of the TypeOne™ Restriction Inhibitor, which in the end proved to be effective for our transformation.

Plasmids pKLN59 and prpF-Km differed significantly in their characteristics, as they contain *X. fastidiosa* or an *E. coli* origin of replication (*oriC*), respectively. However, the fact that both DD*gfp* and DD*rpfF^-^* mutants were obtained only in the presence of a type I R–M inhibitor, which interferes with the cleavage of unmodified DNA, remarks the critical role of these systems in modulating the transformation frequency.

Attempts to exploit the natural competence of the strain De Donno, i.e., the bacterium ability to uptake the DNA from the external environment, failed, confirming that *X. fastidiosa* strains are differently prone to genetic manipulation [26,37]. Natural competence consists in the entrance of extracellular DNA inside the cell, and, once inside the cytoplasm, in the recombination of the foreign DNA into the genome, leading to genetic diversity. Several investigations have demonstrated, for many Gram-negative bacteria, a correlation between natural competence and twitching motility, which depends on components of type IV pili [38,79,80]. In particular, Kung and Almeida [27] showed that the disruption of genes *pilY1*, *pilB*, *pilO,* and *pilQ* involved in the type IV pili biogenesis, affected transformation, and recombination efficiencies. Similarly, analysis of the fringe width in several *X. fastidiosa* strains showed that strains lacking natural competence (BBI64 and Georgia Plum) were also deficient in twitching motility with undetectable peripheral fringe around colonies grown on PD3 plates [37].

Our results support previous findings, showing that the failure of the natural transformation of the strain De Donno was associated with failure to move by twitching as assessed by the lack of fringe around the colonies of the olive strain on PW and PD3 agar plates. In contrast, the evident fringe crown was observed around colonies of the strain Temecula1 consistent with the remarkable potential of this strain to undergo natural competence and recombination [37]. Here we provided evidence that the strain De Donno has some amino acid substitutions, predicted as potentially deleterious when compared to the sequence of Temecula1, in the genes *comA*, *pilA-1,* and *pilM*. This finding suggests that these variations could contribute to the absence of natural competence and the lack of peripheral fringe formation we observed in vitro.

Twitching movement, which enables the upstream migration and the host colonization, is strain-dependent. The reason for this is not yet clear, but it depends on several factors including the number of pili, their length, and the rate of extension and retraction. In addition to long pili, *X. fastidiosa* also exhibits short type I pili, mostly involved in cell and surface attachment and biofilm formation, which may also affect the twitching motility rate, since the adhesion to the surface through type I pili together with the expression of adhesins and hemagglutinin-like proteins could slow down displacement via type IV pili.

The results we obtained from the in vitro growth of the two *X. fastidiosa* strains showed that the strain De Donno, which did not show fringe on agar plates, produced significantly more biofilm than the strain Temecula1. The latter, on the other hand, exhibited a significantly higher amount of cells in the planktonic phase than the olive strain, suggesting an enhanced growth rate in this medium. In accordance with the increased biofilm formation, the cell-to-cell attachment, measured as the settling rate of bacterial aggregates in culture suspension, revealed that the strain De Donno showed a more aggregative phenotype than Temecula1. While investigating some aspects of biofilm physiology in *X. fastidiosa* De Donno, Cattò et al. [46] described this behavior as a hyperattachment phenotype, showing that biofilm detachment was not enhanced even when the strain was exposed to sublethal doses of reducing agents, such as N-acetylcysteine (NAC). However, it should be pointed out that the concentration used in this study was much lower than what was known to disrupt the biofilm of *X. fastidiosa* subsp. *pauca* strain 9a5c, and of other bacterial pathogens [81].

The balance, in the plant, between an exploratory (non-adhesive) and an insect-acquirable (adhesive) phase is the core of the *X. fastidiosa* virulence and its host colonization process [9]. Therefore, it will be of interest to explore whether this hyperadhesive phenotype might significantly influences De Donno virulence. In general, the infection mechanism is complex as it is the result of the expression of a portion of the bacterial genome that is flexible among strains and subspecies [82] and of the plant microenvironment [83,84]. Previous studies have shown that the chemical compositions of the growth media and xylem constituents impact *X. fastidiosa* aggregation, biofilm formation, and planktonic growth [85,86,87].

Our study did not allow us to establish whether the behavior observed in vitro reflects what happens in the plant. However, interestingly, our findings suggesting that *X. fastidiosa* De Donno was not very adept at twitching, are consistent with the pathogenicity trials conducted in different olive cultivars showing the presence of the pathogen in the roots of infected plants, as early as twelve months after pin-prick inoculation of the pathogen into the stem [4].

In conclusion, the availability of mutants of one of the most virulent strains of *X. fastidiosa* paves the way for new investigations, aiming to elucidate host–pathogen interactions underpinning the mechanisms of pathogenicity and the differential responses recorded upon the infection of different olive cultivars [4,88,89,90,91,92], necessary for the implementation of strategies to mitigate the impact of OQDS.

## Figures and Tables

**Table 1:** Primers used in this study.

Name	Sequence 5′-3′ ^a^	Target	Amplicon Size (bp)
Up_F	CATTGACAGGAGACAGAAAGA	Upstream region of *rpfF*	827
Up_R	GCAACACCTTCTTCACGAGGCAGACTGTTGTTCTCCGTAATAGTAGTC		
Down_F	GAGATTTTGAGACACAACGTGGCTTACTCAAAGCTGTGCTGATG	Downstream region of *rpfF*	819
Down_R	GAGTGCTGGTTGCTGATG		
Kan_F	GTCTGCCTCGTGAAG	Kanamycin resistance gene	1203
Kan_R	AAGCCACGTTGTGT		
RpfF_F	ATGTCCGCTGTACAT CCCATTCCT	*rpfF* gene	803
RpfF_R	GCGCTCCATAGTTCGGAGTGATTT		
RpfFMut_F	GAAGCGGACATTAGCGTTAC	Recombination region in DD*rpfF*- mutant	1697 (1370 ^b^)
RpfFMut_R	GCTCGGTCATCTTGGTTTAATG		
Int_F	TAGGGGGTCATCGTGACTTGC	Integration site in DD*gfp* mutant	750
Int_R	CCTCGAGCAAGACGTTTCCC		

^a^ Underlining indicates the 5′ extended region of the primer that is homologous to the antibiotic resistance gene to facilitate fusion with the chromosomal sequence by overlap extension PCR. ^b^ Amplicon size in the wild-type strain with no insertion of the marker gene.

## Supplementary Materials

The following are available online at https://www.mdpi.com/2076-2607/8/11/1832/s1. Table S1: In silico prediction of the effects of sequence variations in genes involved in natural competence and twitching motility.

## References

[1] European Food Safety Authority (2020). Update of the Xylella spp. host plant database–systematic literature search up to 30 June 2019. EFSA J..

[2] Almeida R.P.P. (2016). Xylella fastidiosa vector transmission biology. Vector-Mediated Transmission of Plant Pathogens.

[3] Hopkins D.L., Purcell A.H. (2002). *Xylella fastidiosa*: Cause of Pierce’s disease of grapevine and other emergent diseases. Plant Dis..

[4] Saponari M., Boscia D., Altamura G., Loconsole G., Zicca S., D’Attoma G., Morelli M., Palmisano F., Saponari A., Tavano D. (2017). Isolation and pathogenicity of *Xylella fastidiosa* associated to the olive quick decline syndrome in southern Italy. Sci. Rep..

[5] Saponari M., Giampetruzzi A., Loconsole G., Boscia D., Saldarelli P. (2019). *Xylella fastidiosa* in olive in Apulia: Where we stand. Phytopathology.

[6] Schneider K., Van der Werf W., Cendoya M., Mourits M., Navas-Cortés J.A., Vicent A., Lansink A.O. (2020). Impact of *Xylella fastidiosa* subspecies pauca in European olives. Proc. Natl. Acad. Sci. USA.

[7] Hopkins D.L. (1989). *Xylella fastidiosa*: Xylem-limited bacterial pathogen of plants. Annu. Rev. Phytopathol..

[8] Tyson G.E., Stojanovic B.J., Kuklinski R.F., DiVittorio T.J., Sullivan M.L. (1985). Scanning electron microscopy of Piercés disease bacterium in petiolar xylem of grape leaves. Phytopathology.

[9] Chatterjee S., Newman K.L., Lindow S.E. (2008). Cell-to-cell signaling in *Xylella fastidiosa* suppresses movement and xylem vessel colonization in grape. Mol. Plant-Microbe Interact..

[10] Sun Q., Sun Y., Walker M.A., Labavitch J.M. (2013). Vascular occlusions in grapevines with Pierce’s disease make disease symptom development worse. Plant Physiol..

[11] Newman K.L., Almeida R.P.P., Purcell A.H., Lindow S.E. (2004). Cell-cell signaling controls *Xylella fastidiosa* interactions with both insects and plants. Proc. Natl. Acad. Sci. USA.

[12] Beaulieu E.D., Ionescu M., Chatterjee S., Yokota K., Trauner D., Lindow S. (2013). Characterization of a diffusible signaling factor from *Xylella fastidiosa*. mBio.

[13] Wang N., Li J.-L., Lindow S.E. (2012). RpfF-dependent regulon of *Xylella fastidiosa*. Phytopathology.

[14] Stevenson J.F., Matthews M.A., Greve L.C., Labavitch J.M., Rost T.L. (2004). Grapevine susceptibility to Pierce’s disease II: Progression of anatomical symptoms. Am. J. Enol. Vitic..

[15] Tyree M.T., Zimmermann M.H. (2002). Hydraulic architecture of woody shoots. Xylem Structure and the Ascent of Sap.

[16] Meng Y., Li Y., Galvani C.D., Hao G., Turner J.N., Burr T.J., Hoch H. (2005). Upstream migration of *Xylella fastidiosa* via pilus-driven twitching motility. J. Bacteriol..

[17] Mattick J.S. (2002). Type IV pili and twitching motility. Annu. Rev. Microbiol..

[18] Huang B., Whitchurch C.B., Mattick J.S. (2003). FimX, a multidomain protein connecting environmental signals to twitching motility in Pseudomonas aeruginosa. J. Bacteriol..

[19] Liu H., Kang Y., Genin S., Schell M.A., Denny T.P. (2001). Twitching motility of Ralstonia solanacearum requires a type IV pilus system. Microbiology.

[20] Feil H., Feil W.S., Lindow S.E. (2007). Contribution of fimbrial and afimbrial adhesins of *Xylella fastidiosa* to attachment to surfaces and virulence to grape. Phytopathology.

[21] Guilhabert M.R., Kirkpatrick B.C. (2005). Identification of *Xylella fastidiosa* antivirulence genes: Hemagglutinin adhesins contribute to X. fastidiosa biofilm maturation and colonization and attenuate virulence. Mol. Plant-Microbe Interact..

[22] Caserta R., Takita M., Targon M., Rosselli-Murai L., De Souza A., Peroni L., Stach-Machado D., Andrade A., Labate C., Kitajima E. (2010). Expression of *Xylella fastidiosa* fimbrial and afimbrial proteins during biofilm formation. Appl. Environ. Microbiol..

[23] Danhorn T., Fuqua C. (2007). Biofilm formation by plant-associated bacteria. Annu. Rev. Microbiol..

[24] Cruz L.F., Cobine P.A., De La Fuente L. (2012). Calcium increases *Xylella fastidiosa* surface attachment, biofilm formation, and twitching motility. Appl. Environ. Microbiol..

[25] Sicard A., Zeilinger A.R., Vanhove M., Schartel T.E., Beal D.J., Daugherty M.P., Almeida R.P. (2018). *Xylella fastidiosa*: Insights into an emerging plant pathogen. Annu. Rev. Phytopathol..

[26] Kung S.H., Almeida R.P. (2011). Natural competence and recombination in the plant pathogen *Xylella fastidiosa*. Appl. Environ. Microbiol..

[27] Kung S.H., Almeida R.P. (2014). Biological and genetic factors regulating natural competence in a bacterial plant pathogen. Microbiology.

[28] Thomas C.M., Nielsen K.M. (2005). Mechanisms of, and barriers to, horizontal gene transfer between bacteria. Nat. Rev. Microbiol..

[29] Potnis N., Kandel P.P., Merfa M.V., Retchless A.C., Parker J.K., Stenger D.C., Almeida R.P., Bergsma-Vlami M., Westenberg M., Cobine P.A. (2019). Patterns of inter-and intrasubspecific homologous recombination inform eco-evolutionary dynamics of *Xylella fastidiosa*. ISME J..

[30] Almeida R.P., Nascimento F.E., Chau J., Prado S.S., Tsai C.-W., Lopes S.A., Lopes J.R. (2008). Genetic structure and biology of *Xylella fastidiosa* strains causing disease in citrus and coffee in Brazil. Appl. Environ. Microbiol..

[31] Scally M., Schuenzel E.L., Stouthamer R., Nunney L. (2005). Multilocus sequence type system for the plant pathogen *Xylella fastidiosa* and relative contributions of recombination and point mutation to clonal diversity. Appl. Environ. Microbiol..

[32] Baltrus D.A., Guillemin K., Phillips P.C. (2008). Natural transformation increases the rate of adaptation in the human pathogen Helicobacter pylori. Evol. Int. J. Org. Evol..

[33] Nunney L., Hopkins D.L., Morano L.D., Russell S.E., Stouthamer R. (2014). Intersubspecific recombination in *Xylella fastidiosa* strains native to the United States: Infection of novel hosts associated with an unsuccessful invasion. Appl. Environ. Microbiol..

[34] Nunney L., Schuenzel E.L., Scally M., Bromley R.E., Stouthamer R. (2014). Large-scale intersubspecific recombination in the plant-pathogenic bacterium *Xylella fastidiosa* is associated with the host shift to mulberry. Appl. Environ. Microbiol..

[35] Nunney L., Yuan X., Bromley R.E., Stouthamer R. (2012). Detecting genetic introgression: High levels of intersubspecific recombination found in *Xylella fastidiosa* in Brazil. Appl. Environ. Microbiol..

[36] Friesen T.L., Stukenbrock E.H., Liu Z., Meinhardt S., Ling H., Faris J.D., Rasmussen J.B., Solomon P.S., McDonald B.A., Oliver R.P. (2006). Emergence of a new disease as a result of interspecific virulence gene transfer. Nat. Genet..

[37] Kandel P.P., Almeida R.P.P., Cobine P.A., De La Fuente L. (2017). Natural competence rates are variable among *Xylella fastidiosa* strains and homologous recombination occurs in vitro between subspecies fastidiosa and multiplex. Mol. Plant-Microbe Interact..

[38] Kandel P.P., Lopez S.M., Almeida R.P.P., De La Fuente L. (2016). Natural competence of *Xylella fastidiosa* occurs at a high frequency inside microfluidic chambers mimicking the bacterium’s natural habitats. Appl. Environ. Microbiol..

[39] Kung S.H., Retchless A.C., Kwan J.Y., Almeida R.P. (2013). Effects of DNA size on transformation and recombination efficiencies in *Xylella fastidiosa*. Appl. Environ. Microbiol..

[40] Roberts R.J., Vincze T., Posfai J., Macelis D. (2007). REBASE—enzymes and genes for DNA restriction and modification. Nucleic Acids Res..

[41] Moreira L.M., De Souza R.F., Digiampietri L.A., Da Silva A.C., Setubal J.C. (2005). Comparative analyses of Xanthomonas and Xylella complete genomes. OMICS..

[42] Niza B., Merfa M.V., Alencar V.C., Menegidio F.B., Nunes L.R., Machado M.A., Takita M.A., de Souza A.A. (2016). Draft genome sequence of 11399, a transformable citrus-pathogenic strain of *Xylella fastidiosa*. Genome Announc..

[43] Kobayashi I. (2001). Behavior of restriction–modification systems as selfish mobile elements and their impact on genome evolution. Nucleic Acids Res..

[44] Guilhabert M.R., Kirkpatrick B.C. (2003). Transformation of *Xylella fastidiosa* with broad host range RSF1010 derivative plasmids. Mol. Plant Pathol..

[45] Román Ecija M., Landa B.B., Navas Cortés J.A., Gómez L., Fuente L. Phenotypic characterization of two Spanish strains of Xylella fastidiosa subsp. multiplex ST6 differing in plasmid content. Proceedings of the 2nd European Conference on Xylella fastidiosa (How Research Can Support Solutions).

[46] Cattò C., De Vincenti L., Cappitelli F., D’Attoma G., Saponari M., Villa F., Forlani F. (2019). Non-Lethal Effects of N-Acetylcysteine on *Xylella fastidiosa* Strain De Donno Biofilm Formation and Detachment. Microorganisms.

[47] Giampetruzzi A., D’Attoma G., Zicca S., Abou Kubaa R., Rizzo D., Boscia D., Saldarelli P., Saponari M. (2019). Draft genome sequence resources of three strains (TOS4, TOS5, and TOS14) of *Xylella fastidiosa* infecting different host plants in the newly discovered outbreak in Tuscany, Italy. Phytopathology.

[48] Van Sluys M.A., De Oliveira M.C., Monteiro-Vitorello C.B., Miyaki C.Y., Furlan L.R., Camargo L.E.A., Da Silva A.C.R., Moon D.H., Takita M.A., Lemos E.G.M. (2003). Comparative analyses of the complete genome sequences of Pierce’s disease and citrus variegated chlorosis strains of *Xylella fastidiosa*. J. Bacteriol..

[49] Giampetruzzi A., Saponari M., Almeida R.P.P., Essakhi S., Boscia D., Loconsole G., Saldarelli P. (2017). Complete genome sequence of the olive-infecting strain *Xylella fastidiosa* subsp. pauca De Donno. Genome Announc..

[50] Davis M., Davis M.J., Thomson S.V. (1980). Isolation media for the Pierce’s disease bacterium. Phytopathology.

[51] Davis M.J., French W.J., Schaad N.W. (1981). Axenic culture of the bacteria associated with phony disease of peach and plum leaf scald. Curr. Microbiol..

[52] Allaire J. (2012). RStudio: Integrated development environment for R. Bostonma.

[53] McDonald J.H. (2009). Handbook of Biological Statistics.

[54] Wickham H. (2011). ggplot2. Wiley Interdiscip. Rev. Comput. Stat..

[55] Newman K.L., Almeida R.P.P., Purcell A.H., Lindow S.E. (2003). Use of a green fluorescent strain for analysis of *Xylella fastidiosa* colonization of Vitis vinifera. Appl. Environ. Microbiol..

[56] Kandel P.P., Chen H., De La Fuente L. (2018). A short protocol for gene knockout and complementation in *Xylella fastidiosa* shows that one of the type IV pilin paralogs (PD1926) is needed for twitching while another (PD1924) affects pilus number and location. Appl. Environ. Microbiol..

[57] Wells J.M., Raju B.C., Nyland G., Lowe S.K. (1981). Medium for isolation and growth of bacteria associated with plum leaf scald and phony peach diseases. Appl. Environ. Microbiol..

[58] Loconsole G., Boscia D., Palmisano F., Savino V., Potere O., Martelli G.P., Saponari M. (2014). A *Xylella fastidiosa* strain with unique biology and phylogeny is associated with a severe disease of olive in Southern Apulia. J. Plant Pathol..

[59] Ye J., Coulouris G., Zaretskaya I., Cutcutache I., Rozen S., Madden T.L. (2012). Primer-BLAST: A tool to design target-specific primers for polymerase chain reaction. Bmc Bioinform..

[60] REBASE. The Restriction Enzyme Database. http://rebase.neb.com/rebase/rebase.html.

[61] Ensembl Bacteria. https://bacteria.ensembl.org/index.html.

[62] Altschul S.F., Madden T.L., Schäffer A.A., Zhang J., Zhang Z., Miller W., Lipman D.J. (1997). Gapped BLAST and PSI-BLAST: A new generation of protein database search programs. Nucleic Acids Res..

[63] PROVEAN (Protein Variation Effect Analyzer). provean.jcvi.org.

[64] Choi Y., Chan A.P. (2015). PROVEAN web server: A tool to predict the functional effect of amino acid substitutions and indels. Bioinformatics.

[65] Stoodley P., Cargo R., Rupp C.J., Wilson S., Klapper I. (2002). Biofilm material properties as related to shear-induced deformation and detachment phenomena. J. Ind. Microbiol. Biotechnol..

[66] Janissen R., Murillo D.M., Niza B., Sahoo P.K., Nobrega M.M., Cesar C.L., Temperini M.L., Carvalho H.F., De Souza A.A., Cotta M.A. (2015). Spatio-temporal distribution of different extracellular polymeric substances and filamentation mediate *Xylella fastidiosa* adhesion and biofilm formation. Sci. Rep..

[67] Marques L., Ceri H., Manfio G., Reid D., Olson M. (2002). Characterization of biofilm formation by *Xylella fastidiosa* in vitro. Plant Dis..

[68] Matsumoto A., Igo M.M. (2010). Species-specific type II restriction-modification system of *Xylella fastidiosa* Temecula1. Appl. Environ. Microbiol..

[69] O’ Leary M., Burbank L., Stenger D.C. Distinct genetic lineages of *Xylella fastidiosa* carry conserved Type I Restriction-Modification systems with diverse specificity subunits. Proceedings of the APS Annual Meeting “Plant Health 2020”.

[70] Goosens V.J., Busch A., Georgiadou M., Castagnini M., Forest K.T., Waksman G., Pelicic V. (2017). Reconstitution of a minimal machinery capable of assembling periplasmic type IV pili. Proc. Natl. Acad. Sci. USA.

[71] De La Fuente L., Montanes E., Meng Y., Li Y., Burr T.J., Hoch H., Wu M. (2007). Assessing adhesion forces of type I and type IV pili of *Xylella fastidiosa* bacteria by use of a microfluidic flow chamber. Appl. Environ. Microbiol..

[72] Saponari M., Boscia D., Del Castillo B.B.L., Jacques M.A., Marco E., Poliakoff F. (2018). Emerge of *Xylella fastidiosa* in Europe. Phytopathology.

[73] Giampetruzzi A., Velasco-Amo M.P., Marco-Noales E., Montes-Borrego M., Roman-Ecija M., Navarro I., Monterde A., Barbé S., Almeida R.P.P., Saldarelli P. (2019). Draft genome resources of two strains (“ESVL” and “IVIA5901”) of *Xylella fastidiosa* associated with almond leaf scorch disease in Alicante, Spain. Phytopathology.

[74] Landa B.B., Velasco-Amo M.P., Marco-Noales E., Olmo D., López M.M., Navarro I., Monterde A., Barbé S., Montes-Borrego M., Román-Écija M. (2018). Draft genome sequence of *Xylella fastidiosa* subsp. fastidiosa strain IVIA5235, isolated from Prunus avium in Mallorca Island, Spain. Microbiol. Resour. Announc..

[75] Gomila M., Moralejo E., Busquets A., Segui G., Olmo D., Nieto A., Juan A., Lalucat J. (2019). Draft genome resources of two strains of *Xylella fastidiosa* XYL1732/17 and XYL2055/17 isolated from Mallorca vineyards. Phytopathology.

[76] Della Coletta-Filho H., Takita M.A., de Souza A.A., Aguilar-Vildoso C.I., Machado M.A. (2001). Differentiation of strains of *Xylella fastidiosa* by a variable number of tandem repeat analysis. Appl. Environ. Microbiol..

[77] Loenen W.A., Dryden D.T., Raleigh E.A., Wilson G.G. (2014). Type I restriction enzymes and their relatives. Nucleic Acids Res..

[78] Waldron D.E., Lindsay J.A. (2006). Sau1: A novel lineage-specific type I restriction-modification system that blocks horizontal gene transfer into Staphylococcus aureus and between S. aureus isolates of different lineages. J. Bacteriol..

[79] Chen I., Dubnau D. (2004). DNA uptake during bacterial transformation. Nat. Rev. Microbiol..

[80] Seitz P., Blokesch M. (2013). Cues and regulatory pathways involved in natural competence and transformation in pathogenic and environmental Gram-negative bacteria. FEMS Microbiol. Rev..

[81] Muranaka L.S., Giorgiano T.E., Takita M.A., Forim M.R., Silva L.F., Coletta-Filho H.D., Machado M.A., de Souza A.A. (2013). N-Acetylcysteine in agriculture, a novel use for an old molecule: Focus on controlling the plant–pathogen *Xylella fastidiosa*. PLoS ONE.

[82] Nunes L.R., Rosato Y.B., Muto N.H., Yanai G.M., da Silva V.S., Leite D.B., Gonçalves E.R., de Souza A.A., Coletta-Filho H.D., Machado M.A. (2003). Microarray analyses of *Xylella fastidiosa* provide evidence of coordinated transcription control of laterally transferred elements. Genome Res..

[83] Leite B., Andersen P.C., Ishida M.L. (2004). Colony aggregation and biofilm formation in xylem chemistry-based media for *Xylella fastidiosa*. FEMS Microbiol. Lett..

[84] Bi J., Dumenyo C., Hernandez-Martinez R., Cooksey D., Toscano N. (2007). Effect of host plant xylem fluid on growth, aggregation, and attachment of *Xylella fastidiosa*. J. Chem. Ecol..

[85] Andersen P.C., Brodbeck B.V., Oden S., Shriner A., Leite B. (2007). Influence of xylem fluid chemistry on planktonic growth, biofilm formation and aggregation of *Xylella fastidiosa*. FEMS Microbiol. Lett..

[86] Zaini P.A., De La Fuente L., Hoch H.C., Burr T.J. (2009). Grapevine xylem sap enhances biofilm development by *Xylella fastidiosa*. FEMS Microbiol. Lett..

[87] Cogan N., Donahue M., Whidden M., De La Fuente L. (2013). Pattern formation exhibited by biofilm formation within microfluidic chambers. Biophys. J..

[88] Giampetruzzi A., Morelli M., Saponari M., Loconsole G., Chiumenti M., Boscia D., Savino V.N., Martelli G.P., Saldarelli P. (2016). Transcriptome profiling of two olive cultivars in response to infection by the CoDiRO strain of *Xylella fastidiosa* subsp. pauca. BMC Genom..

[89] De Pascali M., Vergine M., Sabella E., Aprile A., Nutricati E., Nicolì F., Buja I., Negro C., Miceli A., Rampino P. (2019). Molecular effects of *Xylella fastidiosa* and drought combined stress in olive trees. Plants.

[90] Sabella E., Luvisi A., Aprile A., Negro C., Vergine M., Nicolì F., Miceli A., De Bellis L. (2018). *Xylella fastidiosa* induces differential expression of lignification related-genes and lignin accumulation in tolerant olive trees cv. Leccino. J. Plant Physiol..

[91] Saponari M., Altamura G., Abou Kubaa R., Montilon V., Saldarelli P., Specchia F., Palmisano F., Silletti M.R., Pollastro P., Zicca S. Further acquisition on the response of a large number of olive cultivars to infections caused by Xylella fastidiosa subsp. pauca, ST53. Proceedings of the 2nd European Conference on Xylella fastidiosa (How Research Can Support Solutions).

[92] Giampetruzzi A., Baptista P., Morelli M., Cameirão C., Lino Neto T., Costa D., D’Attoma G., Abou Kubaa R., Altamura G., Saponari M. (2020). Differences in the Endophytic Microbiome of Olive Cultivars Infected by *Xylella fastidiosa* across Seasons. Pathogens.

